# Estimated Disease Progression Trajectory of White Matter Disruption in Unilateral Temporal Lobe Epilepsy: A Data-Driven Machine Learning Approach

**DOI:** 10.3390/brainsci14100992

**Published:** 2024-09-29

**Authors:** Daichi Sone, Noriko Sato, Yoko Shigemoto, Iman Beheshti, Yukio Kimura, Hiroshi Matsuda

**Affiliations:** 1 Department of Radiology, National Center of Neurology and Psychiatry, Tokyo 187-8551, Japan; snoriko@ncnp.go.jp (N.S.); yokos@ncnp.go.jp (Y.S.); yukio-k01@ncnp.go.jp (Y.K.); hiroshi.matsuda@mt.strins.or.jp (H.M.); 2 Department of Psychiatry, Jikei University School of Medicine, Tokyo 105-8461, Japan; 3 Department of Human Anatomy and Cell Science, Rady Faculty of Health Sciences, Max Rady College of Medicine, University of Manitoba, Winnipeg, MB R3E 0J9, Canada; iman.beheshti@umanitoba.ca

**Keywords:** temporal lobe epilepsy, white matter, diffusion tensor imaging, machine learning

## Abstract

Background/Objectives: Although the involvement of progressive brain alterations in epilepsy was recently suggested, individual patients’ trajectories of white matter (WM) disruption are not known. Methods: We investigated the disease progression patterns of WM damage and its associations with clinical metrics. We examined the cross-sectional diffusion tensor imaging (DTI) data of 155 patients with unilateral temporal lobe epilepsy (TLE) and 270 age/gender-matched healthy controls, and we then calculated the average fractional anisotropy (FA) values within 20 WM tracts of the whole brain. We used the Subtype and Stage Inference (SuStaIn) program to detect the progression trajectory of FA changes and investigated its association with clinical parameters including onset age, disease duration, drug-responsiveness, and the number of anti-seizure medications (ASMs). Results: The SuStaIn algorithm identified a single subtype model in which the initial damage occurs in the ipsilateral uncinate fasciculus (UF), followed by damage in the forceps, superior longitudinal fasciculus (SLF), and anterior thalamic radiation (ATR). This pattern was replicated when analyzing TLE with hippocampal sclerosis (n = 50) and TLE with no lesions (n = 105) separately. Further-progressed stages were associated with longer disease duration (*p* < 0.001) and a greater number of ASMs (*p* = 0.001). Conclusions: the disease progression model based on WM tracts may be useful as a novel individual-level biomarker.

## 1. Introduction

Epilepsy is a common chronic neurological disorder and is characterized by recurrent seizures caused by abnormal and excessive neural activities [1]. The psychosocial and economic burdens of epilepsy on patients and their caregivers are significant [2,3]. In light of these serious conditions, epilepsy was selected as the target of the World Health Organization’s Intersectional Global Action Plan in 2022 [4]. In fact, problems in epilepsy care include not only seizure control, but also comorbidities and psychosocial issues [5]. To address these complex issues, various advanced biomarkers, including brain imaging, are expected to be developed [6].

In recent years, the disease progression of epilepsy has been a matter of controversy. It is well known that brain atrophy and white matter (WM) damage in epilepsy can extend beyond the epileptogenic foci [7,8], and it has also been suggested that abnormal brain networks are involved in such neuronal damage [9]. A 2019 study using longitudinal brain MRI data showed that in individuals with epilepsy, the rate of cortical thinning over time is higher than that in healthy aging [10]. However, even if epilepsy is progressive, not all patients progress uniformly, and it is not clear in what order the damage progresses. In this regard, estimating the pattern of disease progression in each patient may lead to the development of novel individual-level biomarkers for epilepsy.

To address this issue, another study applying brain morphology MRI reported that the use of an unsupervised machine learning analysis, i.e., the Subtype and Stage Inference (SuStaIn) algorithm [11,12], has made it possible to classify the progressive subtypes and stages of individual brain atrophy in epilepsy [13,14]. In the study, the patterns of brain morphological changes in patients with focal epilepsy were classified into three subtypes: the cortical type, starting with reduced cortical thickness; the basal ganglia type, starting with basal ganglia atrophy; and the hippocampal type, starting with hippocampal atrophy; the hippocampal type was reported to be the most frequent in temporal lobe epilepsy (TLE) [13].

**Figure 1:**
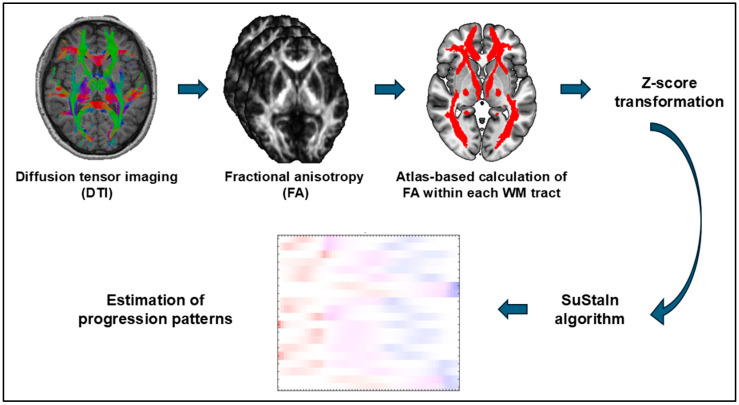
The flow of analysis in this study. The DTI data were processed by tract-based spatial statistics (TBSS) and atlas-based calculation of fractional anisotropy (FA) within each WM tract. The Z-scores were analyzed by SuStaIn algorithm to estimate disease progression patterns.

TLE is the most prevalent form of focal epilepsy and is often refractory to drug treatment [15]. Not only is brain morphology atrophy known to occur in TLE, but so is WM microstructural damage [16]. Since brain morphological alterations are expected to progress in TLE, we hypothesized that WM tract damage may also be progressive along with some specific trajectories. In addition, given the role of WM tracts in connecting different brain regions and the recent concept of epilepsy as a brain network disorder [17], the patterns of WM damage progression could be highly relevant. We speculated that the subtyping and staging of the progression of WM damage over time in TLE may be clinically useful as an individual-level biomarker for categorizing and monitoring disease progression. We thus conducted the present study to identify the subtypes and staging patterns of WM microstructural alterations in TLE, using diffusion tensor imaging (DTI) and data-driven machine learning algorithms. The SuStaIn algorithm was applied to DTI data in 155 unilateral TLE patients and it estimated the progression trajectories of WM disruption. The flow of analysis is shown in Figure 1. We further discussed the potential utilities of the subtyping and staging as a novel individual-level imaging biomarker.

## 2. Materials and Methods

### 2.1. Subjects

We recruited 155 patients with unilateral TLE who were examined at our epilepsy center in Tokyo, Japan between December 2013 and March 2017. Board-certified epileptologists made the diagnosis of TLE based on (i) the presence of focal seizures consistent with TLE, and (ii) focal epileptiform discharge predominantly in unilateral temporal areas as revealed by conventional scalp electroencephalography (EEG). Long-term video-EEG monitoring and/or interictal ^18^F-FDG PET were also performed when needed. High-resolution MRI scans of all patients were visually inspected by experienced neuroradiologists.

Patients with the following criteria were excluded: those with a significant medical history of acute encephalitis, meningitis, severe head trauma, or ischemic encephalopathy; suspicious epileptogenic lesions (e.g., tumor, cortical dysplasia or vascular malformation) on MRI other than ipsilateral hippocampal sclerosis (HS) at the abnormal EEG side; or epileptic paroxysms in extra-temporal regions on EEG.

Two hundred seventy age/gender-matched healthy controls (HCs) without any history of neurological or psychiatric disorders and any use of central nervous system medication were also recruited. All of the subjects provided written informed consent to participate in accordance with the Declaration of Helsinki. This study was approved by the Institutional Review Board at National Center of Neurology and Psychiatry Hospital, Tokyo, Japan.

### 2.2. MRI Acquisitions

All subjects underwent 3.0-T MRI scans with a 32-channel coil (Philips Medical System, Best, The Netherlands). The parameters of the 3D T1-weighted image were the following: repetition time (TR), 7.12 ms; echo time (TE) 3.4 ms; flip angle, 10°; number of excitations (NEX), 1; effective slice thickness, 0.6 mm with no gap; slices, 300; matrix, 260 × 320; and field of view (FOV), 26 × 24 cm. The DTI sequence was obtained with the following parameters: TR, 6700 ms; TE) 58 ms; flip angle, 90°; NEX, 2; effective slice thickness, 3.0 mm with no gap; slices, 60; matrix, 80 × 78; and FOV, 24 × 24 cm. The DTI was acquired along 15 non-collinear directions with a diffusion-weighted b-factor of 1000 s/mm^2^, and one image was acquired without a diffusion gradient. Coronal fluid-attenuated inversion recovery (FLAIR) imaging and transverse 2D turbo spin echo T2-weighted imaging were also obtained for visual inspection.

### 2.3. MRI Processing

The DTI data were initially preprocessed with tract-based spatial statistics (TBSS) with the use of the PANDA toolbox v.1.3.1 (https://www.nitrc.org/projects/panda/ (accessed on 20 January 2023)) [18] running on MATLAB (MathWorks, Natick, MA, USA) and the FMRIB Software Library (FSL) ver. 5.0.11. Eddy current correction and brain extraction were performed, and then the TBSS pipeline provided an atlas-based region-of-interest (ROI) analysis using all tracts of the Johns Hopkins University (JHU) atlas. The automated ROI locations were visually checked for anatomical accuracy. The FA threshold for the TBSS was set at 0.20. The pipeline calculated mean FA values within each tract of the atlas in each patient [19]. We visually confirmed no problematic error or artifact on the quality of the raw and processed DTI data.

### 2.4. Subtype and Stage Inference (SuStaIn) Analysis

First, all of the mean FA values within each tract were corrected for age and sex using a linear regression model as in our previous study [20]. As the SuStaIn algorithm requires Z-scores for the machine learning analysis [11], we calculated Z-scores for each tract’s FA values of the patients by using the data of the 270 healthy controls. Since WM damage in TLE is known to be more profound on the focus side [8], we investigated the WM changes in consideration of the focal side; to analyze left and right TLE together, we reclassified the Z-score of each tract to the ipsilateral and contralateral sides, except for the midline structures, i.e., major and minor forceps.

The Z-scores of all 20 ROIs of the 155 patients with unilateral TLE were entered into the SuStaIn algorithm (https://github.com/ucl-pond/SuStaInMatlab (accessed on 20 January 2023)) as described in our previous study [20]. Although an excessive number of biomarkers may cause problems in this analysis, we considered 20 ROIs would be acceptable based on similar previous studies [20,21]. As a SuStaIn analysis performs an unsupervised machine learning strategy, any information other than the Z-scores, e.g., the anatomy of each ROI or clinical data, was not taken into account. The linear Z-score model and mathematical model underlying the SuStaIn algorithm have been described [11]; the steps include model-fitting, convergence, uncertainty estimation, cross-validation, and similarity between subtypes. As described [11,21,22], the SuStaIn algorithm categorized our individual patients into subtypes and estimated the most likely sequence in which the selected ROIs reached different progression stages over time. While each subject’s stage was estimated as probability values of weighted staging, we utilized the stage with the maximum likelihood as the subject’s progression stage. The optimal number of subtypes was estimated using the cross-validation information criterion (CVIC) to balance model complexity [11,13].

### 2.5. Separate Analyses for the TLE Patients with and without Hippocampal Sclerosis

Although our primary analysis aimed to identify progression patterns in TLE both with and without HS, there could be differences between these two categories, and we therefore separately performed additional SuStaIn analyses for the 50 TLE patients with HS (TLE-HS) and the 105 TLE patients without HS (i.e., TLE with no visible lesions [TLE-NL]).

### 2.6. Statistical Analyses

The Shapiro–Wilk test revealed non-parametric distributions for most of the clinical continuous variables in this study. We investigated the relationships of the disease subtypes and stages derived from the SuStaIn analysis with the following clinical data: focus side, onset age, disease duration, presence of HS, number of antiseizure medications (ASMs), and pharmaco-resistance. We used the χ^2^ test for categorical data, the Mann–Whitney U-test for group comparisons with continuous variables, and Spearman’s rank test for the correlation analysis. A *p*-value < 0.05 was deemed significant. The statistical analyses were performed by SPSS software ver. 25.0 (IBM Corp., Armonk, NY, USA).

## 3. Results

### 3.1. Clinical Demographics

The demographic data of the patients with TLE and the HCs are summarized in Table 1. There was no significant difference in age or sex between the TLE and HC groups. Compared to the TLE-NL patients, the TLE-HS patients had younger onset ages and longer durations of disease, and they used a greater number of ASMs.

### 3.2. SuStaIn Algorithm Results

**Figure 2:**
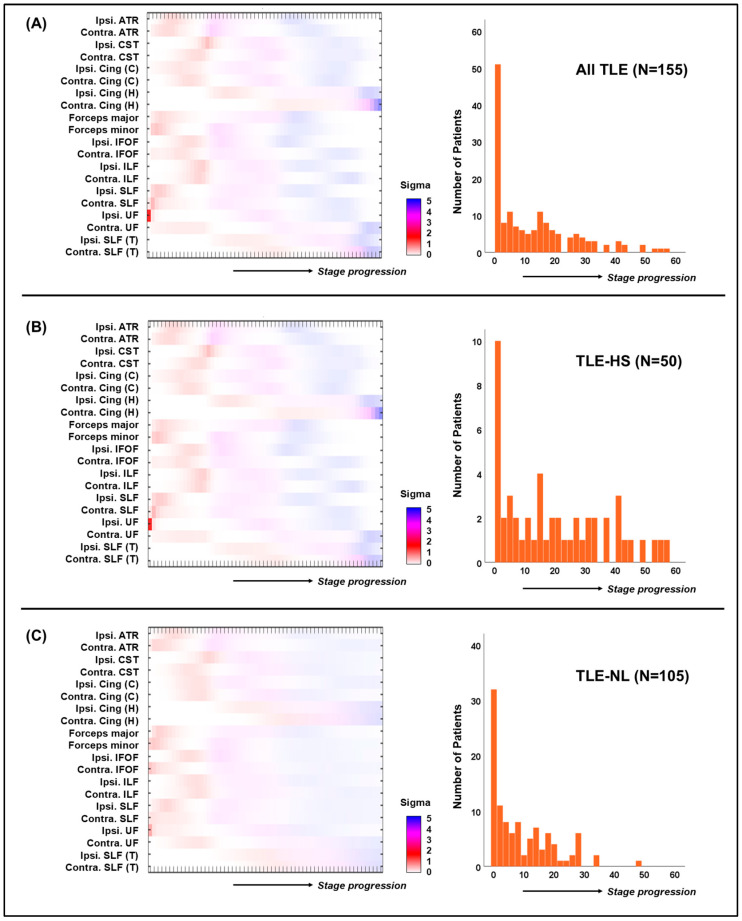
The progression pattern of white matter (WM) tract disruption in temporal lobe epilepsy (TLE) (left) and the number of patients at each progression stage. Results of (**A**) all 155 patients with TLE, (**B**) the 50 patients with TLE with hippocampal sclerosis (HS), and (**C**) the 105 patients with TLE with no visible lesions. ATR: anterior thalamic radiation, CST: corticospinal tract, Cing (C): cingulum (cingulate gyrus), Cing (H): cingulum hippocampus, IFOF: inferior fronto-occipital fasciculus, ILF, inferior longitudinal fasciculus, SLF: superior longitudinal fasciculus, UF: uncinate fasciculus, SLF (T): superior longitudinal fasciculus (temporal projection).

The SuStaIn algorithm identified a single subtype from the WM tract-based mean FA data of the 155 patients with unilateral TLE. In the progression model of this subtype, the initial damage occurs in the ipsilateral uncinate fasciculus (UF), followed by damage in the forceps, superior longitudinal fasciculus (SLF), and anterior thalamic radiation (ATR) (Figure 2A). The cingulum, inferior longitudinal fasciculus (ILF), inferior fronto-occipital fasciculus (IFOF), and corticospinal tract would be disrupted in the middle disease stages.

### 3.3. Associations with Clinical Parameters

**Figure 3:**
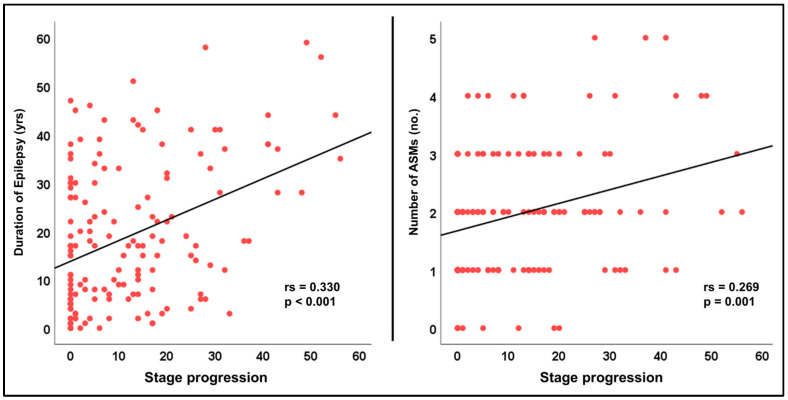
Significant correlations of stage progression with disease duration (**left**) and the number of antiseizure medications (ASMs) used (**right**).

As the SuStaIn algorithm detected just one subtype, we analyzed the relationships between the staging results and clinical parameters (Table 2). We observed that the staging was not significantly associated with gender, side of focus, or seizure freedom. The TLE-HS group showed significantly more progressed stages than the TLE-NL group (*p* < 0.001). Stage progression was also correlated with the disease duration and the number of ASMs (Figure 3).

### 3.4. Separate Analyses for the TLE-HS and TLE-NL Patients

Similar progression patterns were reproduced by the separate analyses for the patients with TLE-HS (Figure 2B) and those with TLE-NL (Figure 2C). In both analyses, one subtype was identified by the SuStaIn algorithm, in which the ipsilateral UF was damaged first and the forceps, SLF, and ATR were damaged at later timepoints (Figure 2B,C). Regarding the clinical associations with staging, similar results, i.e., correlations with disease duration, were observed (Supplement Table S1).

## 4. Discussion

We calculated the progression models of WM damage in patients with TLE, using an unsupervised machine learning algorithm. As a result, the SuStaIn algorithm identified a single subtype in which the ipsilateral UF damage occurred first, and the forceps, SLF, and ATR were damaged subsequently. Since the UF is a part of the limbic system, connecting the anterior temporal lobe and the orbitofrontal cortex [23], our findings are consistent with the anatomical pathophysiology in TLE. This progression pattern model was replicated in the separate analyses for the TLE-HS and TLE-NL groups, indicating that WM changes in TLE may share a similar progression trajectory. Regarding the clinical correlates, further-progressed stages were associated with longer disease durations and the use of a greater number of antiseizure medications. In addition, the patients with TLE with HS showed more advanced stages compared to the TLE patients with no lesions. Although many epilepsy neuroimaging studies have used machine learning, most were supervised learning studies using clinically labeled data, with few reports of unsupervised learning [24]. The advantage of unsupervised learning is that it can be used to find hidden patterns in unlabeled data that are difficult to notice clinically and may thus lead to new discoveries [24].

The white matter damage in TLE is extensive [8,16], but it has not been known when and in what order this damage occurs. The progression of WM disruption over time in TLE has not yet been clearly demonstrated with the use of longitudinal data. However, a 2019 longitudinal morphological MRI study demonstrated that the progression of brain atrophy over time in focal epilepsy exceeds that of normal aging [10], and it is conceivable that white matter damage may also progress over time. The WM damage progression pattern model in TLE that was identified in our present investigation can be used to identify the disease progression stages in individual patients and may serve as a novel clinical biomarker. WM is the structure that communicates between brain regions and serves as the base of the brain network, and has potential for a variety of future studies, which may include epilepsy types other than TLE, relevance to clinical outcomes such as postsurgical seizure freedom, or associations with brain network metrics.

Xiao et al. investigated disease progression patterns of brain atrophy in focal epilepsy and idiopathic generalized epilepsy (IGE) by using cross-sectional MRI data and the SuStaIn algorithm [13]. According to their findings, although IGE presented two different trajectories, i.e., the basal ganglia atrophy type and the cortical thinning type, the brain morphological changes in focal epilepsy were classified into three subtypes: the cortical type, starting with reduced cortical thickness; the basal ganglia type, starting with basal ganglia atrophy; and the hippocampal type, starting with hippocampal atrophy; in addition, the hippocampal type was reported to be the most frequent in TLE [13]. Our present analyses identified only one subtype for WM progression, possibly because we selected a relatively homogeneous clinical group, i.e., patients with unilateral TLE. Another possible explanation might be the use of a tract-level evaluation. Using tract-based mean FA values alone may not assess white matter damage in sufficient anatomical detail and might warrant further investigation using a better methodology beyond a tract-level analysis. Conversely, if only one subtype actually exists, a more specific method for time-based modeling, rather than the spatiotemporal heterogeneity approach [25], may be useful for further detailed investigation.

We also detected several clinical correlates with disease progression stages. The TLE-HS patients presented more progressed stages compared to the TLE-NL patients, and this may reflect more severe WM damage in the TLE-HS group. It has been repeatedly confirmed that the integrity of the white matter in individuals with TLE-HS is more profoundly impaired [8,16]. We also observed a positive correlation between staging and disease duration (Spearman’s rs = 0.330, *p* < 0.001), which is consistent with the recent study using morphological brain MRI [13]. In TLE, both gray matter atrophy and WM fiber damage may progress over time along with the duration of disease. The number of ASMs used may also be an important factor affecting WM disruption. As our cohort was mostly drug-resistant cases, caution should be used when considering the nonsignificant results between staging and seizure freedom, considering the small sample of seizure-free patients. In addition, due to the cross-sectional design, causal relationships between these associations cannot be addressed. We did not investigate the effect of seizure burden. While no significant correlations between disease stages and seizure frequency were found in the previous study [13], further investigations would be warranted for these issues.

This study has several limitations. The sample size was medium (155 patients with TLE and 270 healthy controls) from a single epilepsy center, and careful interpretation would be needed for sub-analyses with a small sample size, e.g., seizure-free patients (N = 14 in total). This study lacked external validation, although the results were generally replicated by the additional analyses performed separately for the TLE-HS and TLE-NL groups. It should also be noted that our findings are based solely on cross-sectional data and theoretical models, and thus our results must be tested in studies with larger cohorts and longitudinal investigations. Our clinical data were also limited, lacking more detailed examinations, e.g., cognitive dysfunction or surgical outcomes. More detailed clinical data could be useful in the future to further explore the potential utility of SuStaIn results as a clinical biomarker. There might be other unknown or unevaluated confounders, e.g., the effect of medications, which should be considered for careful interpretations of the results of this study.

## 5. Conclusions

Using a data-driven machine learning analysis, we identified the white matter disease progression trajectory in patients with unilateral TLE, in which the initial damage occurs in the ipsilateral UF, followed by damage in the forceps, SLF, and ATR. More progressed stages of TLE were associated with the presence of hippocampal sclerosis, longer disease duration, and a greater number of ASMs used. These findings may contribute to the better pathophysiological understanding of the progression of temporal lobe epilepsy as well as the establishment of novel imaging biomarkers.

## Figures and Tables

**Table 1:** Demographics of the patients with temporal lobe epilepsy and the healthy controls.

	TLE	HC	*p*-Value	TLE-HS	TLE-NL	*p*-Value
N	155	270	NA	50	105	NA
Age (yrs) median (IQR)	42 (26)	45 (16)	0.354	44 (21)	40 (27)	0.997
Gender (M:F)	68:87	119:151	0.968	18:32	50:55	0.173
Onset age (yrs) median (IQR)	20 (22)	NA	NA	10 (15)	24 (30)	<0.001
Duration (yrs) median (IQR)	17 (24)	NA	NA	28 (20)	9 (19)	<0.001
Laterality	L = 107, R = 48	NA	NA	L = 32, R = 18	L = 75, R = 30	0.35
Etiology	HS = 50, NL = 105	NA	NA	NA	NA	NA
Number of ASMs median (IQR) *	2 (2)	NA	NA	2 (1)	2 (1)	0.002
Seizure freedom	SF = 14, not SF = 141	NA	NA	SF = 2, not SF = 48	SF = 12, not SF = 93	0.131

TLE: temporal lobe epilepsy, HC: healthy controls, HS: hippocampal sclerosis, NA: not available, NL: no lesion. ASMs: antiseizure medications, SF: seizure freedom. * missing in 5 patients.

**Table 2:** Associations between progression stages and clinical parameters in patients with temporal lobe epilepsy.

Categorical Comparison		
Categories and median (IQR) Stages		*p*-value
Male	Female	
5.5 (18)	9 (21)	0.382
HS	NL	
16 (28)	5 (15)	<0.001
Left TLE	Right TLE	
7 (17)	11.5 (24)	0.205
SF	not SF	
6.5 (13)	8 (19)	0.427
**Correlation analysis**		
Parameters	Spearman’s rs	*p*-value
Age	0.102	0.207
Onset age	−0.191	0.017
Duration	0.330	<0.001
Number of ASMs	0.269	0.001

HS: hippocampal sclerosis, NL: no lesion, TLE: temporal lobe epilepsy, SF: seizure freedom, ASMs: antiseizure medications.

## Data Availability

Data not included in the article will be made available from the corresponding author to qualified researchers on reasonable request subject to ethics approval.

## Supplementary Materials

The following supporting information can be downloaded at: https://www.mdpi.com/article/10.3390/brainsci14100992/s1, Table S1: Associations between progression stages and clinical parameters derived from the separate analyses for the TLE-HS and TLE-NL groups.
