# Supplementary material for: Estimated Disease Progression Trajectory of White Matter Disruption in Unilateral Temporal Lobe Epilepsy: A Data-Driven Machine Learning Approach
Source: Brain Sci. 2024 Sep 29;14(10):992. doi: 10.3390/brainsci14100992 (PMC11506697; doi:10.3390/brainsci14100992)
Supplement: Supplementary file 1 [file brainsci-14-00992-s001.zip › brainsci-3197654-supplementary.pdf]

**Supplementary Table S1.** Associations between progression stages and clinical parameters derived from the separate analyses for the TLE-HS and TLE-NL groups

| **TLE-HS, n=50** | | | **TLE-NL, n=105** | | |
|---|---|---|---|---|---|
| **Categorical comparison:** | | | **Categorical comparison:** | | |
| Categories and median (IQR) stages | | p-value | Categories and median IQR stages | | p-value |
| Male | Female | | Male | Female | |
| 16 (28) | 16 (31) | 0.984 | 4 (15) | 7 (15) | 0.515 |
| Left TLE | Right TLE | | Left TLE | Right TLE | |
| 15 (30) | 18.5 (29) | 0.478 | 4 (14) | 6.5 (16) | 0.485 |
| SF | not SF | | SF | not SF | |
| 21 (NA) | 16 (27) | >0.999 | 6.5 (12) | 4 (16) | 0.814 |
| **Correlation analysis:** | | | **Correlation analysis:** | | |
| Parameter | Spearman's rs | p-value | Parameter | Spearman's rs | p-value |
| Age | 0.253 | 0.076 | Age | 0.052 | 0.599 |
| Onset age | −0.074 | 0.609 | Onset age | −0.075 | 0.445 |
| Duration | 0.377 | 0.007 | Duration | 0.215 | 0.028 |
| No. of ASMs | 0.187 | 0.198 | No. of ASMs | 0.238 | 0.017 |

ASMs: antiseizure medications, HS: hippocampal sclerosis, NL: no lesion, SF: seizure freedom, TLE: temporal lobe epilepsy.
